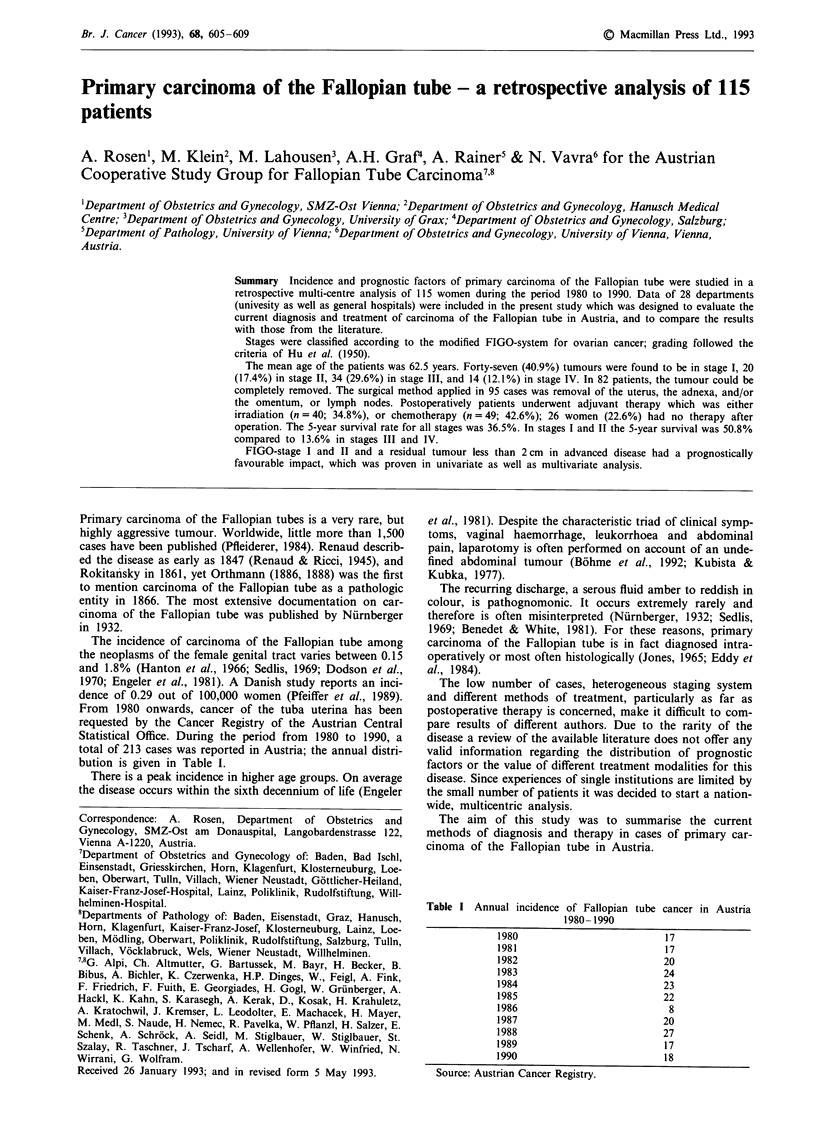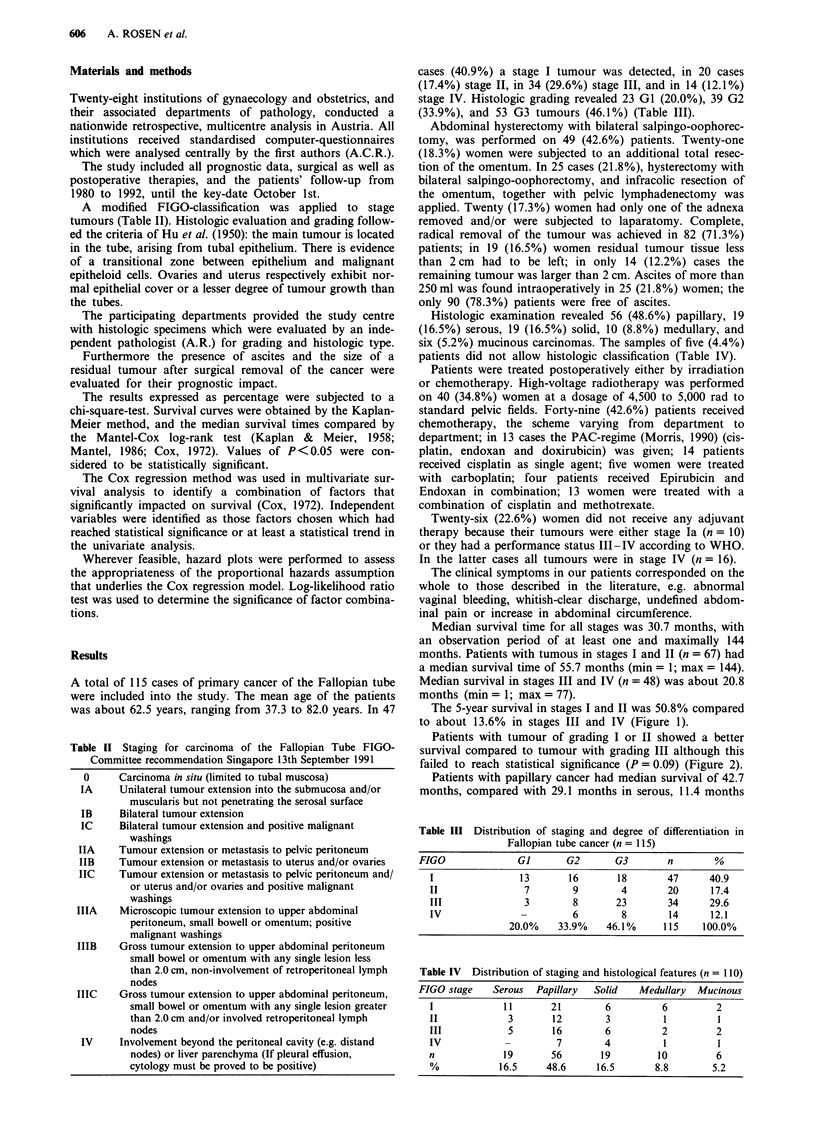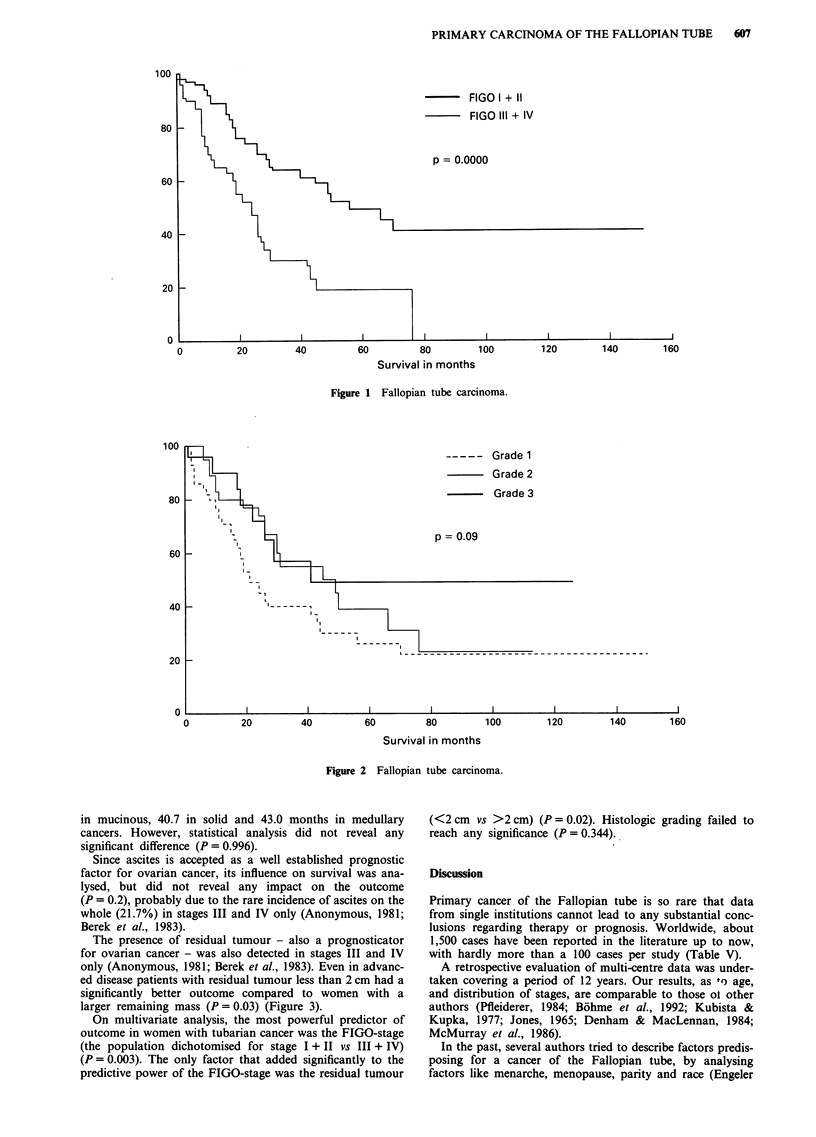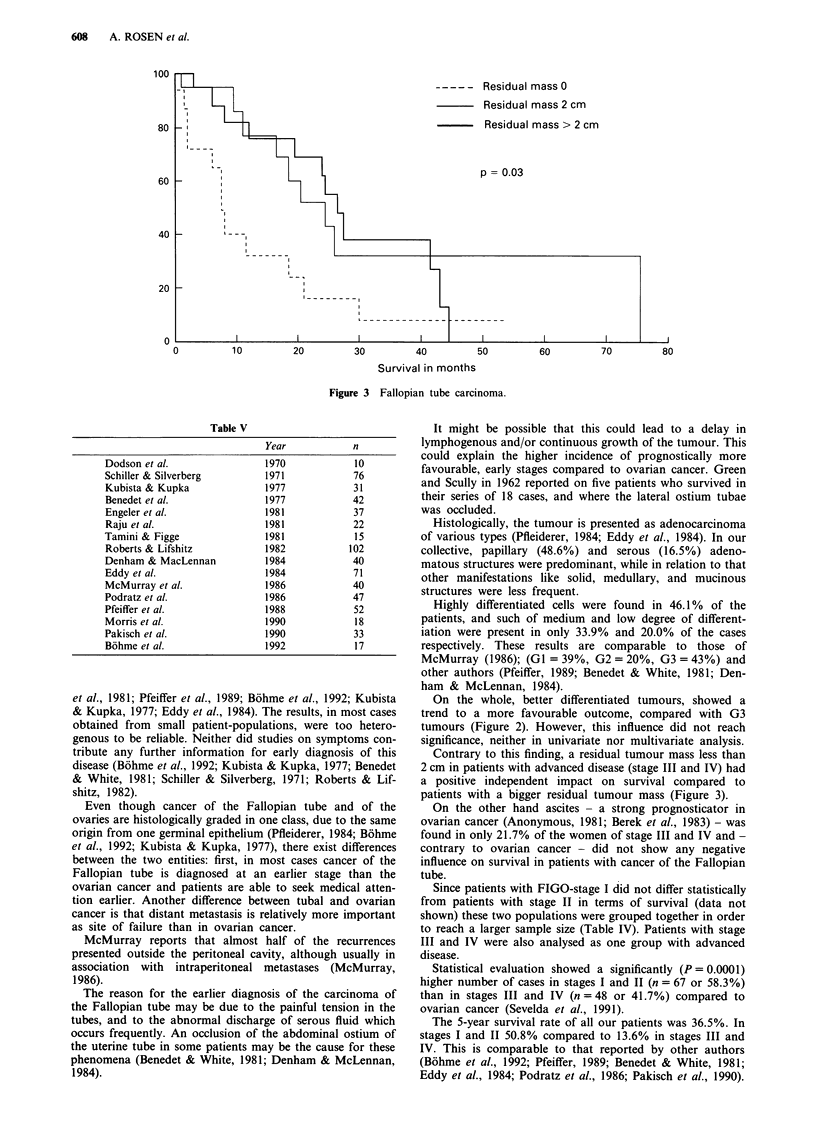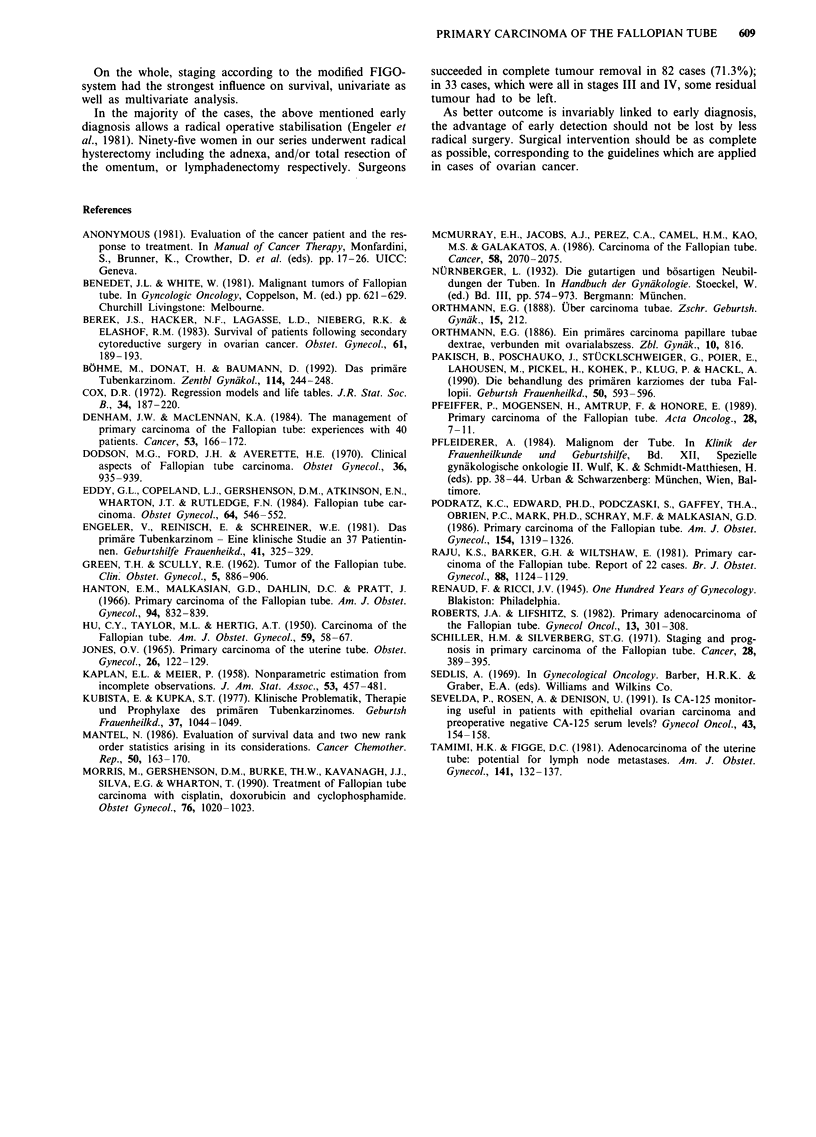# Primary carcinoma of the fallopian tube--a retrospective analysis of 115 patients. Austrian Cooperative Study Group for Fallopian Tube Carcinoma.

**DOI:** 10.1038/bjc.1993.394

**Published:** 1993-09

**Authors:** A. Rosen, M. Klein, M. Lahousen, A. H. Graf, A. Rainer, N. Vavra

**Affiliations:** Department of Obstetrics and Gynecology, SMZ-Ost Vienna, Austria.

## Abstract

Incidence and prognostic factors of primary carcinoma of the Fallopian tube were studied in a retrospective multi-centre analysis of 115 women during the period 1980 to 1990. Data of 28 departments (university as well as general hospitals) were included in the present study which was designed to evaluate the current diagnosis and treatment of carcinoma of the Fallopian tube in Austria, and to compare the results with those from the literature. Stages were classified according to the modified FIGO-system for ovarian cancer; grading followed the criteria of Hu et al. (1950). The mean age of the patients was 62.5 years. Forty-seven (40.9%) tumours were found to be in stage I, 20 (17.4%) in stage II, 34 (29.6%) in stage III, and 14 (12.1%) in stage IV. In 82 patients, the tumour could be completely removed. The surgical method applied in 95 cases was removal of the uterus, the adnexa, and/or the omentum, or lymph nodes. Postoperatively patients underwent adjuvant therapy which was either irradiation (n = 40; 34.8%), or chemotherapy (n = 49; 42.6%); 26 women (22.6%) had no therapy after operation. The 5-year survival rate for all stages was 36.5%. In stages I and II the 5-year survival was 50.8% compared to 13.6% in stages III and IV. FIGO-stage I and II and a residual tumour less than 2 cm in advanced disease had a prognostically favourable impact, which was proven in univariate as well as multivariate analysis.


*Br. J. Cancer* (1993), **68**, 605–609 © Macmillan Press Ltd., 1993

### Primary carcinoma of the Fallopian tube – a retrospective analysis of 115 patients

A. Rosen[1], M. Klein[2], M. Lahousen[3], A.H. Graf[4], A. Rainer[5] & N. Vavra[6] for the Austrian Cooperative Study Group for Fallopian Tube Carcinoma[7,8]

[1]*Department of Obstetrics and Gynecology, SMZ-Ost Vienna;* [2]*Department of Obstetrics and Gynecoloyg, Hanusch Medical Centre;* [3]*Department of Obstetrics and Gynecology, University of Grax;* [4]*Department of Obstetrics and Gynecology, Salzburg;* [5]*Department of Pathology, University of Vienna;* [6]*Department of Obstetrics and Gynecology, University of Vienna, Vienna, Austria.*

**Summary** Incidence and prognostic factors of primary carcinoma of the Fallopian tube were studied in a retrospective multi-centre analysis of 115 women during the period 1980 to 1990. Data of 28 departments (univesity as well as general hospitals) were included in the present study which was designed to evaluate the current diagnosis and treatment of carcinoma of the Fallopian tube in Austria, and to compare the results with those from the literature.

Stages were classified according to the modified FIGO-system for ovarian cancer; grading followed the criteria of Hu *et al.* (1950).

The mean age of the patients was 62.5 years. Forty-seven (40.9%) tumours were found to be in stage I, 20 (17.4%) in stage II, 34 (29.6%) in stage III, and 14 (12.1%) in stage IV. In 82 patients, the tumour could be completely removed. The surgical method applied in 95 cases was removal of the uterus, the adnexa, and/or the omentum, or lymph nodes. Postoperatively patients underwent adjuvant therapy which was either irradiation ($n = 40$; 34.8%), or chemotherapy ($n = 49$; 42.6%); 26 women (22.6%) had no therapy after operation. The 5-year survival rate for all stages was 36.5%. In stages I and II the 5-year survival was 50.8% compared to 13.6% in stages III and IV.

FIGO-stage I and II and a residual tumour less than 2 cm in advanced disease had a prognostically favourable impact, which was proven in univariate as well as multivariate analysis.

Primary carcinoma of the Fallopian tubes is a very rare, but highly aggressive tumour. Worldwide, little more than 1,500 cases have been published (Pfleiderer, 1984). Renaud described the disease as early as 1847 (Renaud & Ricci, 1945), and Rokitansky in 1861, yet Orthmann (1886, 1888) was the first to mention carcinoma of the Fallopian tube as a pathologic entity in 1866. The most extensive documentation on carcinoma of the Fallopian tube was published by Nürnberger in 1932.

The incidence of carcinoma of the Fallopian tube among the neoplasms of the female genital tract varies between 0.15 and 1.8% (Hanton *et al.*, 1966; Sedlis, 1969; Dodson *et al.*, 1970; Engeler *et al.*, 1981). A Danish study reports an incidence of 0.29 out of 100,000 women (Pfeiffer *et al.*, 1989). From 1980 onwards, cancer of the tuba uterina has been requested by the Cancer Registry of the Austrian Central Statistical Office. During the period from 1980 to 1990, a total of 213 cases was reported in Austria; the annual distribution is given in Table I.

There is a peak incidence in higher age groups. On average the disease occurs within the sixth decennium of life (Engeler *et al.*, 1981). Despite the characteristic triad of clinical symptoms, vaginal haemorrhage, leukorrhoea and abdominal pain, laparotomy is often performed on account of an undefined abdominal tumour (Böhme *et al.*, 1992; Kubista & Kubka, 1977).

The recurring discharge, a serous fluid amber to reddish in colour, is pathognomonic. It occurs extremely rarely and therefore is often misinterpreted (Nürnberger, 1932; Sedlis, 1969; Benedet & White, 1981). For these reasons, primary carcinoma of the Fallopian tube is in fact diagnosed intra-operatively or most often histologically (Jones, 1965; Eddy *et al.*, 1984).

The low number of cases, heterogeneous staging system and different methods of treatment, particularly as far as postoperative therapy is concerned, make it difficult to compare results of different authors. Due to the rarity of the disease a review of the available literature does not offer any valid information regarding the distribution of prognostic factors or the value of different treatment modalities for this disease. Since experiences of single institutions are limited by the small number of patients it was decided to start a nationwide, multicentric analysis.

The aim of this study was to summarise the current methods of diagnosis and therapy in cases of primary carcinoma of the Fallopian tube in Austria.

**Table I** Annual incidence of Fallopian tube cancer in Austria 1980–1990

| | |
|---|---|
| 1980 | 17 |
| 1981 | 17 |
| 1982 | 20 |
| 1983 | 24 |
| 1984 | 23 |
| 1985 | 22 |
| 1986 | 8 |
| 1987 | 20 |
| 1988 | 27 |
| 1989 | 17 |
| 1990 | 18 |

Source: Austrian Cancer Registry.

Correspondence: A. Rosen, Department of Obstetrics and Gynecology, SMZ-Ost am Donauspital, Langobardenstrasse 122, Vienna A-1220, Austria.

[7]Department of Obstetrics and Gynecology of: Baden, Bad Ischl, Einsenstadt, Griesskirchen, Horn, Klagenfurt, Klosterneuburg, Loeben, Oberwart, Tulln, Villach, Wiener Neustadt, Göttlicher-Heiland, Kaiser-Franz-Josef-Hospital, Lainz, Poliklinik, Rudolfstiftung, Willhelminen-Hospital.

[8]Departments of Pathology of: Baden, Eisenstadt, Graz, Hanusch, Horn, Klagenfurt, Kaiser-Franz-Josef, Klosterneuburg, Lainz, Loeben, Mödling, Oberwart, Poliklinik, Rudolfstiftung, Salzburg, Tulln, Villach, Vöcklabruck, Wels, Wiener Neustadt, Willhelminen.

[7,8]G. Alpi, Ch. Altmutter, G. Bartussek, M. Bayr, H. Becker, B. Bibus, A. Bichler, K. Czerwenka, H.P. Dinges, W., Feigl, A. Fink, F. Friedrich, F. Fuith, E. Georgiades, H. Gogl, W. Grünberger, A. Hackl, K. Kahn, S. Karasegh, A. Kerak, D., Kosak, H. Krahuletz, A. Kratochwil, J. Kremser, L. Leodolter, E. Machacek, H. Mayer, M. Medl, S. Naude, H. Nemec, R. Pavelka, W. Pflanzl, H. Salzer, E. Schenk, A. Schröck, A. Seidl, M. Stiglbauer, W. Stiglbauer, St. Szalay, R. Taschner, J. Tscharf, A. Wellenhofer, W. Winfried, N. Wirrani, G. Wolfram.

Received 26 January 1993; and in revised form 5 May 1993.

### Materials and methods

Twenty-eight institutions of gynaecology and obstetrics, and their associated departments of pathology, conducted a nationwide retrospective, multicentre analysis in Austria. All institutions received standardised computer-questionnaires which were analysed centrally by the first authors (A.C.R.).

The study included all prognostic data, surgical as well as postoperative therapies, and the patients' follow-up from 1980 to 1992, until the key-date October 1st.

A modified FIGO-classification was applied to stage tumours (Table II). Histologic evaluation and grading followed the criteria of Hu *et al.* (1950): the main tumour is located in the tube, arising from tubal epithelium. There is evidence of a transitional zone between epithelium and malignant epitheloid cells. Ovaries and uterus respectively exhibit normal epithelial cover or a lesser degree of tumour growth than the tubes.

The participating departments provided the study centre with histologic specimens which were evaluated by an independent pathologist (A.R.) for grading and histologic type.

Furthermore the presence of ascites and the size of a residual tumour after surgical removal of the cancer were evaluated for their prognostic impact.

The results expressed as percentage were subjected to a chi-square-test. Survival curves were obtained by the Kaplan-Meier method, and the median survival times compared by the Mantel-Cox log-rank test (Kaplan & Meier, 1958; Mantel, 1986; Cox, 1972). Values of $P<0.05$ were considered to be statistically significant.

The Cox regression method was used in multivariate survival analysis to identify a combination of factors that significantly impacted on survival (Cox, 1972). Independent variables were identified as those factors chosen which had reached statistical significance or at least a statistical trend in the univariate analysis.

Wherever feasible, hazard plots were performed to assess the appropriateness of the proportional hazards assumption that underlies the Cox regression model. Log-likelihood ratio test was used to determine the significance of factor combinations.

### Results

A total of 115 cases of primary cancer of the Fallopian tube were included into the study. The mean age of the patients was about 62.5 years, ranging from 37.3 to 82.0 years. In 47 cases (40.9%) a stage I tumour was detected, in 20 cases (17.4%) stage II, in 34 (29.6%) stage III, and in 14 (12.1%) stage IV. Histologic grading revealed 23 G1 (20.0%), 39 G2 (33.9%), and 53 G3 tumours (46.1%) (Table III).

Abdominal hysterectomy with bilateral salpingo-oophorectomy, was performed on 49 (42.6%) patients. Twenty-one (18.3%) women were subjected to an additional total resection of the omentum. In 25 cases (21.8%), hysterectomy with bilateral salpingo-oophorectomy, and infracolic resection of the omentum, together with pelvic lymphadenectomy was applied. Twenty (17.3%) women had only one of the adnexa removed and/or were subjected to laparatomy. Complete, radical removal of the tumour was achieved in 82 (71.3%) patients; in 19 (16.5%) women residual tumour tissue less than 2 cm had to be left; in only 14 (12.2%) cases the remaining tumour was larger than 2 cm. Ascites of more than 250 ml was found intraoperatively in 25 (21.8%) women; the only 90 (78.3%) patients were free of ascites.

Histologic examination revealed 56 (48.6%) papillary, 19 (16.5%) serous, 19 (16.5%) solid, 10 (8.8%) medullary, and six (5.2%) mucinous carcinomas. The samples of five (4.4%) patients did not allow histologic classification (Table IV).

Patients were treated postoperatively either by irradiation or chemotherapy. High-voltage radiotherapy was performed on 40 (34.8%) women at a dosage of 4,500 to 5,000 rad to standard pelvic fields. Forty-nine (42.6%) patients received chemotherapy, the scheme varying from department to department; in 13 cases the PAC-regime (Morris, 1990) (cisplatin, endoxan and doxirubicin) was given; 14 patients received cisplatin as single agent; five women were treated with carboplatin; four patients received Epirubicin and Endoxan in combination; 13 women were treated with a combination of cisplatin and methotrexate.

Twenty-six (22.6%) women did not receive any adjuvant therapy because their tumours were either stage Ia ($n = 10$) or they had a performance status III–IV according to WHO. In the latter cases all tumours were in stage IV ($n = 16$).

The clinical symptoms in our patients corresponded on the whole to those described in the literature, e.g. abnormal vaginal bleeding, whitish-clear discharge, undefined abdominal pain or increase in abdominal circumference.

Median survival time for all stages was 30.7 months, with an observation period of at least one and maximally 144 months. Patients with tumous in stages I and II ($n = 67$) had a median survival time of 55.7 months (min = 1; max = 144). Median survival in stages III and IV ($n = 48$) was about 20.8 months (min = 1; max = 77).

The 5-year survival in stages I and II was 50.8% compared to about 13.6% in stages III and IV (Figure 1).

Patients with tumour of grading I or II showed a better survival compared to tumour with grading III although this failed to reach statistical significance ($P = 0.09$) (Figure 2).

Patients with papillary cancer had median survival of 42.7 months, compared with 29.1 months in serous, 11.4 months

**Table II** Staging for carcinoma of the Fallopian Tube FIGO-Committee recommendation Singapore 13th September 1991

| | |
|---|---|
| 0 | Carcinoma *in situ* (limited to tubal muscosa) |
| IA | Unilateral tumour extension into the submucosa and/or muscularis but not penetrating the serosal surface |
| IB | Bilateral tumour extension |
| IC | Bilateral tumour extension and positive malignant washings |
| IIA | Tumour extension or metastasis to pelvic peritoneum |
| IIB | Tumour extension or metastasis to uterus and/or ovaries |
| IIC | Tumour extension or metastasis to pelvic peritoneum and/ or uterus and/or ovaries and positive malignant washings |
| IIIA | Microscopic tumour extension to upper abdominal peritoneum, small bowell or omentum; positive malignant washings |
| IIIB | Gross tumour extension to upper abdominal peritoneum small bowel or omentum with any single lesion less than 2.0 cm, non-involvement of retroperitoneal lymph nodes |
| IIIC | Gross tumour extension to upper abdominal peritoneum, small bowel or omentum with any single lesion greater than 2.0 cm and/or involved retroperitoneal lymph nodes |
| IV | Involvement beyond the peritoneal cavity (e.g. distand nodes) or liver parenchyma (If pleural effusion, cytology must be proved to be positive) |

**Table III** Distribution of staging and degree of differentiation in Fallopian tube cancer ($n = 115$)

| *FIGO* | *G1* | *G2* | *G3* | *n* | *%* |
|---|---|---|---|---|---|
| I | 13 | 16 | 18 | 47 | 40.9 |
| II | 7 | 9 | 4 | 20 | 17.4 |
| III | 3 | 8 | 23 | 34 | 29.6 |
| IV | – | 6 | 8 | 14 | 12.1 |
| | 20.0% | 33.9% | 46.1% | 115 | 100.0% |

**Table IV** Distribution of staging and histological features ($n = 110$)

| *FIGO stage* | *Serous* | *Papillary* | *Solid* | *Medullary* | *Mucinous* |
|---|---|---|---|---|---|
| I | 11 | 21 | 6 | 6 | 2 |
| II | 3 | 12 | 3 | 1 | 1 |
| III | 5 | 16 | 6 | 2 | 2 |
| IV | – | 7 | 4 | 1 | 1 |
| *n* | 19 | 56 | 19 | 10 | 6 |
| % | 16.5 | 48.6 | 16.5 | 8.8 | 5.2 |

**Figure 1** Fallopian tube carcinoma.

**Figure 2** Fallopian tube carcinoma.

in mucinous, 40.7 in solid and 43.0 months in medullary cancers. However, statistical analysis did not reveal any significant difference ($P = 0.996$).

Since ascites is accepted as a well established prognostic factor for ovarian cancer, its influence on survival was analysed, but did not reveal any impact on the outcome ($P = 0.2$), probably due to the rare incidence of ascites on the whole (21.7%) in stages III and IV only (Anonymous, 1981; Berek *et al.*, 1983).

The presence of residual tumour – also a prognosticator for ovarian cancer – was also detected in stages III and IV only (Anonymous, 1981; Berek *et al.*, 1983). Even in advanced disease patients with residual tumour less than 2 cm had a significantly better outcome compared to women with a larger remaining mass ($P = 0.03$) (Figure 3).

On multivariate analysis, the most powerful predictor of outcome in women with tubarian cancer was the FIGO-stage (the population dichotomised for stage I + II *vs* III + IV) ($P = 0.003$). The only factor that added significantly to the predictive power of the FIGO-stage was the residual tumour (<2 cm *vs* >2 cm) ($P = 0.02$). Histologic grading failed to reach any significance ($P = 0.344$).

### Discussion

Primary cancer of the Fallopian tube is so rare that data from single institutions cannot lead to any substantial conclusions regarding therapy or prognosis. Worldwide, about 1,500 cases have been reported in the literature up to now, with hardly more than a 100 cases per study (Table V).

A retrospective evaluation of multi-centre data was undertaken covering a period of 12 years. Our results, as to age, and distribution of stages, are comparable to those of other authors (Pfleiderer, 1984; Böhme *et al.*, 1992; Kubista & Kupka, 1977; Jones, 1965; Denham & MacLennan, 1984; McMurray *et al.*, 1986).

In the past, several authors tried to describe factors predisposing for a cancer of the Fallopian tube, by analysing factors like menarche, menopause, parity and race (Engeler

**Figure 3** Fallopian tube carcinoma.

**Table V**

| | *Year* | *n* |
|---|---|---|
| Dodson *et al.* | 1970 | 10 |
| Schiller & Silverberg | 1971 | 76 |
| Kubista & Kupka | 1977 | 31 |
| Benedet *et al.* | 1977 | 42 |
| Engeler *et al.* | 1981 | 37 |
| Raju *et al.* | 1981 | 22 |
| Tamini & Figge | 1981 | 15 |
| Roberts & Lifshitz | 1982 | 102 |
| Denham & MacLennan | 1984 | 40 |
| Eddy *et al.* | 1984 | 71 |
| McMurray *et al.* | 1986 | 40 |
| Podratz *et al.* | 1986 | 47 |
| Pfeiffer *et al.* | 1988 | 52 |
| Morris *et al.* | 1990 | 18 |
| Pakisch *et al.* | 1990 | 33 |
| Böhme *et al.* | 1992 | 17 |

*et al.*, 1981; Pfeiffer *et al.*, 1989; Böhme *et al.*, 1992; Kubista & Kupka, 1977; Eddy *et al.*, 1984). The results, in most cases obtained from small patient-populations, were too heterogenous to be reliable. Neither did studies on symptoms contribute any further information for early diagnosis of this disease (Böhme *et al.*, 1992; Kubista & Kupka, 1977; Benedet & White, 1981; Schiller & Silverberg, 1971; Roberts & Lifshitz, 1982).

Even though cancer of the Fallopian tube and of the ovaries are histologically graded in one class, due to the same origin from one germinal epithelium (Pfleiderer, 1984; Böhme *et al.*, 1992; Kubista & Kupka, 1977), there exist differences between the two entities: first, in most cases cancer of the Fallopian tube is diagnosed at an earlier stage than the ovarian cancer and patients are able to seek medical attention earlier. Another difference between tubal and ovarian cancer is that distant metastasis is relatively more important as site of failure than in ovarian cancer.

McMurray reports that almost half of the recurrences presented outside the peritoneal cavity, although usually in association with intraperitoneal metastases (McMurray, 1986).

The reason for the earlier diagnosis of the carcinoma of the Fallopian tube may be due to the painful tension in the tubes, and to the abnormal discharge of serous fluid which occurs frequently. An occlusion of the abdominal ostium of the uterine tube in some patients may be the cause for these phenomena (Benedet & White, 1981; Denham & McLennan, 1984).

It might be possible that this could lead to a delay in lymphogenous and/or continuous growth of the tumour. This could explain the higher incidence of prognostically more favourable, early stages compared to ovarian cancer. Green and Scully in 1962 reported on five patients who survived in their series of 18 cases, and where the lateral ostium tubae was occluded.

Histologically, the tumour is presented as adenocarcinoma of various types (Pfleiderer, 1984; Eddy *et al.*, 1984). In our collective, papillary (48.6%) and serous (16.5%) adenomatous structures were predominant, while in relation to that other manifestations like solid, medullary, and mucinous structures were less frequent.

Highly differentiated cells were found in 46.1% of the patients, and such of medium and low degree of differentiation were present in only 33.9% and 20.0% of the cases respectively. These results are comparable to those of McMurray (1986); (G1 = 39%, G2 = 20%, G3 = 43%) and other authors (Pfeiffer, 1989; Benedet & White, 1981; Denham & McLennan, 1984).

On the whole, better differentiated tumours, showed a trend to a more favourable outcome, compared with G3 tumours (Figure 2). However, this influence did not reach significance, neither in univariate nor multivariate analysis.

Contrary to this finding, a residual tumour mass less than 2 cm in patients with advanced disease (stage III and IV) had a positive independent impact on survival compared to patients with a bigger residual tumour mass (Figure 3).

On the other hand ascites – a strong prognosticator in ovarian cancer (Anonymous, 1981; Berek *et al.*, 1983) – was found in only 21.7% of the women of stage III and IV and – contrary to ovarian cancer – did not show any negative influence on survival in patients with cancer of the Fallopian tube.

Since patients with FIGO-stage I did not differ statistically from patients with stage II in terms of survival (data not shown) these two populations were grouped together in order to reach a larger sample size (Table IV). Patients with stage III and IV were also analysed as one group with advanced disease.

Statistical evaluation showed a significantly ($P = 0.0001$) higher number of cases in stages I and II ($n = 67$ or 58.3%) than in stages III and IV ($n = 48$ or 41.7%) compared to ovarian cancer (Sevelda *et al.*, 1991).

The 5-year survival rate of all our patients was 36.5%. In stages I and II 50.8% compared to 13.6% in stages III and IV. This is comparable to that reported by other authors (Böhme *et al.*, 1992; Pfeiffer, 1989; Benedet & White, 1981; Eddy *et al.*, 1984; Podratz *et al.*, 1986; Pakisch *et al.*, 1990).

On the whole, staging according to the modified FIGO-system had the strongest influence on survival, univariate as well as multivariate analysis.

In the majority of the cases, the above mentioned early diagnosis allows a radical operative stabilisation (Engeler *et al.*, 1981). Ninety-five women in our series underwent radical hysterectomy including the adnexa, and/or total resection of the omentum, or lymphadenectomy respectively. Surgeons succeeded in complete tumour removal in 82 cases (71.3%); in 33 cases, which were all in stages III and IV, some residual tumour had to be left.

As better outcome is invariably linked to early diagnosis, the advantage of early detection should not be lost by less radical surgery. Surgical intervention should be as complete as possible, corresponding to the guidelines which are applied in cases of ovarian cancer.

### References

ANONYMOUS (1981). Evaluation of the cancer patient and the response to treatment. In *Manual of Cancer Therapy*, Monfardini, S., Brunner, K., Crowther, D. *et al.* (eds). pp. 17-26. UICC: Geneva.

BENEDET, J.L. & WHITE, W. (1981). Malignant tumors of Fallopian tube. In *Gyncologic Oncology*, Coppelson, M. (ed.) pp. 621-629. Churchill Livingstone: Melbourne.

BEREK, J.S., HACKER, N.F., LAGASSE, L.D., NIEBERG, R.K. & ELASHOF, R.M. (1983). Survival of patients following secondary cytoreductive surgery in ovarian cancer. *Obstet. Gynecol.*, **61**, 189-193.

BÖHME, M., DONAT, H. & BAUMANN, D. (1992). Das primäre Tubenkarzinom. *Zentbl Gynäkol.*, **114**, 244-248.

COX, D.R. (1972). Regression models and life tables. *J.R. Stat. Soc. B.*, **34**, 187-220.

DENHAM, J.W. & MACLENNAN, K.A. (1984). The management of primary carcinoma of the Fallopian tube: experiences with 40 patients. *Cancer*, **53**, 166-172.

DODSON, M.G., FORD, J.H. & AVERETTE, H.E. (1970). Clinical aspects of Fallopian tube carcinoma. *Obstet Gynecol.*, **36**, 935-939.

EDDY, G.L., COPELAND, L.J., GERSHENSON, D.M., ATKINSON, E.N., WHARTON, J.T. & RUTLEDGE, F.N. (1984). Fallopian tube carcinoma. *Obstet Gynecol.*, **64**, 546-552.

ENGELER, V., REINISCH, E. & SCHREINER, W.E. (1981). Das primäre Tubenkarzinom - Eine klinische Studie an 37 Patientinnen. *Geburtshilfe Frauenheikd.*, **41**, 325-329.

GREEN, T.H. & SCULLY, R.E. (1962). Tumor of the Fallopian tube. *Clin. Obstet. Gynecol.*, **5**, 886-906.

HANTON, E.M., MALKASIAN, G.D., DAHLIN, D.C. & PRATT, J. (1966). Primary carcinoma of the Fallopian tube. *Am. J. Obstet. Gynecol.*, **94**, 832-839.

HU, C.Y., TAYLOR, M.L. & HERTIG, A.T. (1950). Carcinoma of the Fallopian tube. *Am. J. Obstet. Gynecol.*, **59**, 58-67.

JONES, O.V. (1965). Primary carcinoma of the uterine tube. *Obstet. Gynecol.*, **26**, 122-129.

KAPLAN, E.L. & MEIER, P. (1958). Nonparametric estimation from incomplete observations. *J. Am. Stat. Assoc.*, **53**, 457-481.

KUBISTA, E. & KUPKA, S.T. (1977). Klinische Problematik, Therapie und Prophylaxe des primären Tubenkarzinomes. *Geburtsh Frauenheilkd.*, **37**, 1044-1049.

MANTEL, N. (1986). Evaluation of survival data and two new rank order statistics arising in its considerations. *Cancer Chemother. Rep.*, **50**, 163-170.

MORRIS, M., GERSHENSON, D.M., BURKE, TH.W., KAVANAGH, J.J., SILVA, E.G. & WHARTON, T. (1990). Treatment of Fallopian tube carcinoma with cisplatin, doxorubicin and cyclophosphamide. *Obstet Gynecol.*, **76**, 1020-1023.

MCMURRAY, E.H., JACOBS, A.J., PEREZ, C.A., CAMEL, H.M., KAO, M.S. & GALAKATOS, A. (1986). Carcinoma of the Fallopian tube. *Cancer*, **58**, 2070-2075.

NÜRNBERGER, L. (1932). Die gutartigen und bösartigen Neubildungen der Tuben. In *Handbuch der Gynäkologie*. Stoeckel, W. (ed.) Bd. III, pp. 574-973. Bergmann: München.

ORTHMANN, E.G. (1888). Über carcinoma tubae. *Zschr. Geburtsh. Gynäk.*, **15**, 212.

ORTHMANN, E.G. (1886). Ein primäres carcinoma papillare tubae dextrae, verbunden mit ovarialabszess. *Zbl. Gynäk.*, **10**, 816.

PAKISCH, B., POSCHAUKO, J., STÜCKLSCHWEIGER, G., POIER, E., LAHOUSEN, M., PICKEL, H., KOHEK, P., KLUG, P. & HACKL, A. (1990). Die behandlung des primären karziomes der tuba Fallopii. *Geburtsh Frauenheilkd.*, **50**, 593-596.

PFEIFFER, P., MOGENSEN, H., AMTRUP, F. & HONORE, E. (1989). Primary carcinoma of the Fallopian tube. *Acta Oncolog.*, **28**, 7-11.

PFLEIDERER, A. (1984). Malignom der Tube. In *Klinik der Frauenheilkunde und Geburtshilfe*, Bd. XII, Spezielle gynäkologische onkologie II. Wulf, K. & Schmidt-Matthiesen, H. (eds). pp. 38-44. Urban & Schwarzenberg: München, Wien, Baltimore.

PODRATZ, K.C., EDWARD, PH.D., PODCZASKI, S., GAFFEY, TH.A., OBRIEN, P.C., MARK, PH.D., SCHRAY, M.F. & MALKASIAN, G.D. (1986). Primary carcinoma of the Fallopian tube. *Am. J. Obstet. Gynecol.*, **154**, 1319-1326.

RAJU, K.S., BARKER, G.H. & WILTSHAW, E. (1981). Primary carcinoma of the Fallopian tube. Report of 22 cases. *Br. J. Obstet. Gynecol.*, **88**, 1124-1129.

RENAUD, F. & RICCI, J.V. (1945). *One Hundred Years of Gynecology*. Blakiston: Philadelphia.

ROBERTS, J.A. & LIFSHITZ, S. (1982). Primary adenocarcinoma of the Fallopian tube. *Gynecol Oncol.*, **13**, 301-308.

SCHILLER, H.M. & SILVERBERG, ST.G. (1971). Staging and prognosis in primary carcinoma of the Fallopian tube. *Cancer*, **28**, 389-395.

SEDLIS, A. (1969). In *Gynecological Oncology*. Barber, H.R.K. & Graber, E.A. (eds). Williams and Wilkins Co.

SEVELDA, P., ROSEN, A. & DENISON, U. (1991). Is CA-125 monitoring useful in patients with epithelial ovarian carcinoma and preoperative negative CA-125 serum levels? *Gynecol Oncol.*, **43**, 154-158.

TAMIMI, H.K. & FIGGE, D.C. (1981). Adenocarcinoma of the uterine tube: potential for lymph node metastases. *Am. J. Obstet. Gynecol.*, **141**, 132-137.